# Paracrine roles of cellular senescence in promoting tumourigenesis

**DOI:** 10.1038/s41416-018-0066-1

**Published:** 2018-04-19

**Authors:** Jose Mario Gonzalez-Meljem, John Richard Apps, Helen Christina Fraser, Juan Pedro Martinez-Barbera

**Affiliations:** 1 Developmental Biology and Cancer Research Programme, UCL Great Ormond Street Institute of Child Health, Guilford Street, London, WC1N 1EH UK; 2 Basic Research Department, Instituto Nacional de Geriatría, Anillo Periférico 2767, Magdalena Contreras, 10200 Mexico City, Mexico

**Keywords:** Cancer microenvironment, Senescence

## Abstract

Senescent cells activate genetic programmes that irreversibly inhibit cellular proliferation, but also endow these cells with distinctive metabolic and signalling phenotypes. Although senescence has historically been considered a protective mechanism against tumourigenesis, the activities of senescent cells are increasingly being associated with age-related diseases, including cancer. An important feature of senescent cells is the secretion of a vast array of pro-inflammatory cytokines, chemokines, and growth factors collectively known as the senescence-associated secretory phenotype (SASP). Recent research has shown that SASP paracrine signalling can mediate several pro-tumourigenic effects, such as enhancing malignant phenotypes and promoting tumour initiation. In this review, we summarise the paracrine activities of senescent cells and their role in tumourigenesis through direct effects on growth and proliferation of tumour cells, tumour angiogenesis, invasion and metastasis, cellular reprogramming and emergence of tumour-initiating cells, and tumour interactions with the local immune environment. The evidence described here suggests cellular senescence acts as a double-edged sword in cancer pathogenesis, which demands further attention in order to support the use of senolytic or SASP-modulating compounds for cancer treatment.

## Introduction

The field of senescence has greatly expanded since the sensencent cell state was first observed in normal human fibroblasts, by Hayflick and Moorhead, over half a century ago.^1^ Initially referring to the finite proliferative capacity of cells in vitro, senescence is now defined as a cellular state of stable and long-term loss of proliferative capacity, but with the retention of normal metabolic activity and viability. It is characterised by specific changes in morphology (e.g. enlarged and flat cells), metabolism (e.g. increased glycolysis over mitochondrial oxidative phosphorylation), and cell physiology (e.g. resistance to apoptosis).^2–5^

**Fig. 1:**
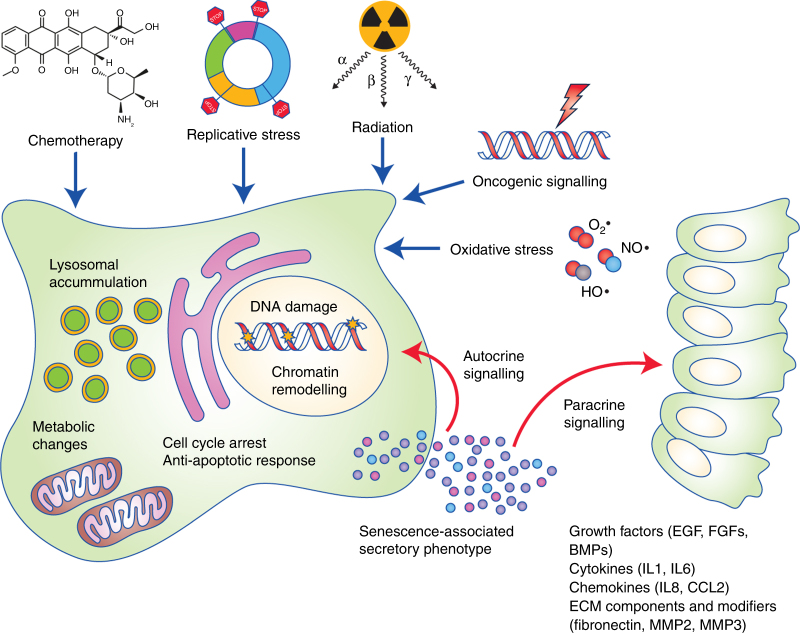
Overview of senescence inducers, changes in cell physiology, and activation of the senescence-associated secretory phenotype (SASP). The senescence programme can be activated by different stress stimuli (shown in blue) such as: cytotoxic chemotherapeutic drugs, replicative stress (which occurs due to deficiencies in the DNA replication machinery or maintenance of cell cycle checkpoints), ionising radiation, oncogenic signalling, and oxidative stress. The main cellular and molecular effects are shown in red and include an expansion of the lysosomal compartment, metabolic and mitochondrial alterations, accumulation of DNA damage and rearrangement of the chromatin landscape, resistance to apoptosis, and an irreversible arrest of the cell cycle. Most senescent cells also activate a senescence-associated secretory phenotype (SASP), which is composed of growth factors, cytokines, chemokines, and metalloproteinases. Examples of common SASP factors are shown. These secreted factors can signal in an autocrine fashion to reinforce the senescence phenotype, or paracrinally with multiple effects on neighbouring cells. EGF epithelial growth factor, FGFs fibroblast growth factors, BMPs bone morphogenetic proteins, IL1 interleukin 1, IL6 interleukin 6, IL8 interleukin 8, CCL2 C–C motif chemokine ligand 2, MMP2 matrix metallopeptidase 2, MMP3 matrix metallopeptidase 3

Senescence serves as a response to stress, and several inducing stimuli have now been identified including chemotherapeutic, radiation and oxidative stress, amongst others (Fig. 1). Activation of the senescence programme leads to cellular and molecular changes such as proliferation arrest, chromatin remodelling, elevated expression of cell cycle inhibitors (such as p16^INK4A^ or p21^CIP1^), activation of a DNA damage response, enlargement of the lysosomal compartment, and activation of a senescence-associated secretory phenotype (SASP).^5,6^ The SASP mediates the paracrine activities of senescent cells through the secretion of a myriad of factors including cytokines and chemokines (e.g. IL1α, IL1β, IL6, IL8, CXCL1, CXCL2), growth factors (e.g. amphiregulin, EGF, BMPs, FGFs, VEGF, WNTs), extracellular matrix (ECM) components (e.g. fibronectin), and proteases (e.g. MMPs, plasminogen activators), as well as exosome-like small extracellular vesicles.^7,3,8–11^ The composition and intensity of the SASP response can be affected by several factors including the senescence-inducing mechanism, cell type, and the amount of time passed since senescence initiation, indicating that there is no singular SASP.^12–17^

SASP effects can be beneficial or deleterious for normal physiology depending on its composition, intensity, and the local tissue microenvironment. Furthermore, the SASP is involved in valuable physiological processes such as promoting tissue repair,^18–20^ fine-tuning the development of embryonic structures,^21–23^ and stimulating immune surveillance.^24,25^ However, the deleterious consequences that result from ineffective clearance of senescent cells and their over-accumulation in tissues can promote age-related diseases and cancer.^2,26–30^ Supporting this notion, the burden of senescent cells in tissues increases significantly with age in mice, primates, and humans,^27^ and they can be found in both benign and malignant tumours.^31–35^ Importantly, genetic or chemical ablation of senescent cells in mouse models delays the onset of age-related disorders, including cancer, leading to increased life-spans and promoting tissue rejuvenation in late life.^36–38^

**Fig. 2:**
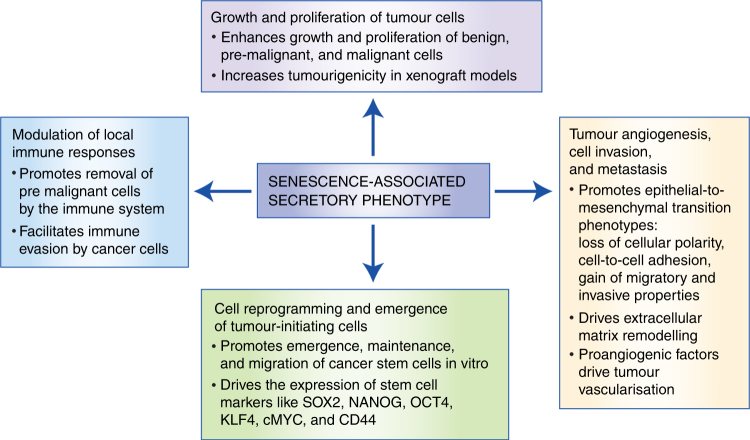
Summary of the paracrine effects of the SASP in promoting tumourigenesis

Senescence was traditionally considered an innate anti-cancer mechanism as it can serve to eliminate damaged cells,^3,5^ whereby activation of the senescence programme in cells harbouring oncogenic mutations serves as a tumour suppressor mechanism, preventing the expansion of these mutated cells and progression into malignancies.^3,5^ However, the role of senescence in tumourigenesis has been revised in recent years. There is mounting evidence that dysregulation or inappropriate activation of senescence contributes to tumour progression and malignancy.^5,7,17^ This review will discuss the paracrine effects of senescent cells on different aspects of tumour cell behaviour including: (i) direct effects on growth and proliferation of tumour cells; (ii) tumour angiogenesis, invasion, and metastasis; (iii) cellular reprogramming and emergence of tumour-initiating cells; and (iv) tumour interactions with the local immune environment (Fig. 2). These subdivisions of the senescence-associated activities are mainly conceptual; senescent cells exert compounded effects and it is not easy to distinguish between some of these activities through current experimental approaches, especially in an in vivo context. For in-depth discussion of the functions of cellular senescence in physiological processes, such as embryological development and tissue repair, as well as in ageing, we refer the reader to other reviews in the field.^2,3^

### Growth and proliferation of tumour cells

Cells present in the tumour microenvironment, such as fibroblasts, can become senescent and promote the growth and proliferation of tumour cells.^7,39,40^ This has been demonstrated both in vitro and in vivo. Co-culture of senescent fibroblasts, induced by various stimuli (e.g. radiation, DNA damage, replicative exhaustion), can promote the growth and proliferation of benign, pre-malignant, and malignant cells from a range of tumour types.^9,12,26,41–44^ For example, in co-culture assays, radiation-induced senescent fibroblasts sustained the growth of mammary epithelial cells that had dysregulated cell cycle and cell death pathways.^44^ These in vitro observations have been further substantiated in vivo, where co-injection of senescent fibroblasts has been shown to increase tumourigenicity in xenograft models, including primary breast cancer tissues.^26,45–47^

The contributions of specific SASP components have been demonstrated using genetic knockdown, siRNAs, and other molecular inhibitors.^12,26,41,45,46,48,49^ The use of siRNA and blocking antibodies against amphiregulin (AREG) reduced the growth of benign prostate epithelial cells induced by conditioned media from senescent fibroblasts.^48^ Furthermore, a critical role for SASP in the promotion of obesity-associated liver cancer has been demonstrated using elegant genetic approaches,^50^ where deletion of IL1β (*Il1b*) was sufficient to reduce the expression of IL6 and CXCL1 in the liver, as well as the number and size of liver tumours.

Finally, there is evidence showing that the expression of growth factors alone, including some that fall under the SASP umbrella, can induce tumours independently in a paracrine/non-cell autonomous manner.^51^ For example, expression of fibroblast growth factor 10 (FGF10) by urogenital mesenchymal cells results in the induction of multifocal prostatic adenocarcinoma in epithelial cells.^52^ Similarly, expression of fibroblast growth factor 19 (FGF19) by skeletal muscle cells has been shown to induce hepatocellular carcinomas, which acquire somatic mutations in β-catenin (*Ctnnb1*).^53^ These experiments collectively demonstrate that the SASP can promote cancer cell growth, challenging the view that senescence is primordially a beneficial process involved in preventing cancer progression.

### Tumour angiogenesis, invasion, and metastasis

Senescent cells can contribute to the acquisition of invasive and metastatic properties of cancer cells, as well as the induction of tumour-associated angiogenesis.^7,39^ Tumour invasion and metastasis frequently involve an epithelial to mesenchymal shift in cellular phenotype (epithelial–mesenchymal transition, EMT). During EMT, epithelial cells attain key aspects enabling tumour invasion, including loss of cellular polarity and cell-to-cell adhesion, and gain of both migratory and invasive properties. Importantly, it is known that conditioned media from senescent cells can induce EMT in cell lines derived from many tumour types, including non-aggressive breast cancer, mesothelioma, and melanoma, as evidenced by decreased expression of epithelial markers (e.g. E-cadherin, cytokeratins) and increased expression of mesenchymal markers (e.g. vimentin).^12,54,55^ In addition, individual SASP components can contribute to induce EMT phenotypes; IL6, for example, has been shown to have cell-adhesion disrupting actions, which is an important component of invasion.^56^ Senescent cells and the SASP can also guide and promote cancer cell migration/invasion in models of thyroid and skin cancers.^57,58^ Furthermore, ablation of senescent cells after chemotherapy can prevent or delay cancer relapse and spread to distal tissues.^59^ Tumour invasion and metastasis also involve disruption of the basement membrane and remodelling of the ECM by matrix metalloproteinases (MMPs), which are often expressed as SASP factors.^7^ Indeed, the invasive properties of several epithelial cell types are enhanced by MMPs secreted by senescent cells, such as MMP2 and MMP3.^41,43,44^

A large number of proangiogenic factors are also known to be secreted by senescent cells, whereas angiostatic molecules have not been found to be secreted.^27,60^ In particular, IL6 has been reported to promote tumour-supportive angiogenesis in a Ras-driven tumour model.^61^ Similarly, co-injection of senescent fibroblasts or peritoneal mesothelial cells with cancer cells in xenograft models results in significantly greater tumour angiogenesis.^62,63^ These data suggest that the paracrine activities of senescent cells are involved in the acquisition of malignant and metastatic phenotypes by signalling to transformed cells or their microenvironment.

### Cellular reprogramming of cells and emergence of tumour-initiating cells in culture

Tumour cells may exhibit loss of differentiation and may also attain stem cell characteristics; both features of cancer progression. In benign tumours and well-differentiated cancers, the histology of a tumour typically recapitulates the histology of the tissue of origin. In contrast, undifferentiated cancers have abnormal histology and typically exhibit more aggressive behaviour, as less differentiated cells are usually more proliferative.

Interestingly, the SASP is able to inhibit differentiation both in vitro and in vivo, while in some cases leads to acquisition of stem cell characteristics.^41,44,54,55,64–66^ Exposure of keratinocytes to the culture medium from senescent cells promotes expression of tumour stem cell markers, such as CD44, and leads to a greater regenerative capacity in vivo*.* ^65^ Similarly, co-culturing undifferentiated myeloma cells in conditioned media from senescent myeloma cells promotes the emergence, maintenance, and migration of cancer stem-like cells.^64^ Higher in vivo expression of stem cell markers has also been observed in the liver in close association with GFP-labelled RAS-induced senescent cells.^65^ In addition, induction of senescence and SASP in mesothelioma cells led to the emergence of a subpopulation of highly clonogenic cells with enhanced ability to form tumours when xenografted in mice.^55^ Furthermore, cellular reprogramming, which is the process by which adult differentiated cells can be induced to become functionally equivalent to embryonic stem cells, can be induced in vivo by senescent cells through SASP activation, and this can be stimulated in different models of tissue damage. While senescence is a barrier to reprogramming in vitro, the paracrine activities of senescent cells can promote the expression of stem cell markers and proliferation of neighbouring cells in vivo*,* ^66–68^ and IL6 is a key player in driving this process.

The molecular mechanisms underpinning the paracrine induction of cancer stem cell features have been variably addressed. For instance, non-tumourigenic melanoma cells exposed to IL6 or chemokine ligand-2 (CCL2) develop tumourigenic potential in vivo in a STAT3-dependent manner.^54^ In vitro, co-culture experiments showed that SASP induced the expression of critical reprogramming factors NANOG, SOX2, and OCT4.^54^ Indeed, it has further been shown that increased IL6 expression, through induction of senescence either genetically or from tissue damage, can create a tissue context that increases reprogramming efficiency in vivo*.* ^66^ In this sense, a crucial role for the mechanistic target of rapamycin (mTOR) complex has recently been unveiled, whereby it can either counteract or facilitate reprogramming by cell-intrinsic and cell-extrinsic mechanisms, respectively.^69^ Together, these data suggest that senescent cells, through their SASP, can induce undifferentiated cellular states; depending on the context, this can be beneficial (e.g. tissue regeneration) or harmful (e.g. promotion of tumour-initiating cells).

### Modulation of local immune response and immune evasion by senescent cells

The relationship between senescence, tumourigenesis, and the immune system is complex and remains incompletely understood. Cells undergoing damage-induced senescence are often cleared by the immune system, as several SASP factors are cytokines and chemokines that can modulate the local immune environment.^2,3,5,70,71^ In this regard, the SASP has been shown to promote inflammation.^7,72^

Immune surveillance refers to the removal of pathogens, as well as pre-malignant and malignant cells, by the immune system. In some cases, it has been shown that senescent cells are involved in these processes. For example, senescent cells promote their own clearance through the secretion of CCL2, which attracts and activates NK-T cells.^73,74^ Using a mouse model of liver carcinoma, p53-deficient RAS-driven tumours induced to senesce through re-establishment of p53 function exhibited innate immune cells migrating into the vicinity of the senescent tumour area, leading to complete tumour regression.^24^ Such senescence-induced activation of the local immune system has also been shown to activate the clearance of pre-malignant hepatocytes.^75^

In contrast, senescent cells can also promote tumour evasion of immune surveillance.^76,77^ During ageing of the skin, senescent stromal cells and their SASP (particularly IL6) drive an increase in the number of suppressive myeloid cells in mice and humans. Furthermore, it was shown that this leads to the inhibition of anti-tumour T-cell responses and enhanced tumour growth.^77^ Further research is required to clarify the factors that control the pro- and anti-tumour surveillance activities of senescent cells.

## Conclusion

There is increasing evidence indicating that, in addition to their cell- and non-cell autonomous tumour-suppressive activities, the paracrine signals derived from senescent cells have detrimental roles in aging-related pathogenesis and cancer. Since senescent cells are generally abundant in benign tumours and also present at low numbers in several malignancies,^31–34,57^ their paracrine activities could contribute to tumour progression and cancer metastasis. Moreover, it is possible that these activities may also be involved in the initial steps of oncogenic transformation of normal cells and tumour initiation, as recently suggested in a mouse model of a human brain tumour.^78^ Promising translational opportunities have emerged in the use of molecules that selectively target and eliminate senescent cells (termed senolytics), or those that modulate the SASP and its negative effects (Table 1).^79^ In this regard, the elimination of senescent cells or targeting the SASP represents a potential strategy for stopping or slowing tumour progression, as many activities of senescent cells promote tumour growth and malignant progression. It may be expected that the same paracrine activities capable of enhancing the cancerous phenotype of cells harbouring oncogenic mutations in vitro and in vivo, could also contribute to the initial epigenetic and genetic alterations that fuel the appearance of tumour-initiating cells in normal, non-transformed cells.^78^ If so, early ablation of senescent cells in pre-malignant lesions using senolytic compounds or neutralisation of the SASP may provide a plausible approach to prevent cancer.

**Table 1:** Examples of compounds that target senescent cells or their SASP

Name	Targets	Mechanism	References
ABT-737	BCL-2 family members	Senolytic	^80^
Navitoclax (ABT-263)	BCL-2 family members	Senolytic	^36^
Metformin	NF-κB pathway members and Dicer	SASP modulator	^81^
Dasatinib	Several tyrosine kinases	Senolytic	^82^
Rapamycin	mTOR	SASP modulator	^46, 49^
Anakinra	IL1 receptor (IL1R)	SASP modulator	^83^
Alvespimycin (17-DMAG)	Heat shock protein 90 (HSP90) chaperone family	Senolytic	^84^
